# High-Frequency Detection of *fosA3* and *bla*_*CTX–M–*55_ Genes in *Escherichia coli* From Longitudinal Monitoring in Broiler Chicken Farms

**DOI:** 10.3389/fmicb.2022.846116

**Published:** 2022-05-18

**Authors:** Maísa Fabiana Menck-Costa, Ana Angelita Sampaio Baptista, Luiz Eduardo de Souza Gazal, Larissa Justino, Matheus Silva Sanches, Marielen de Souza, Erick Kenji Nishio, Beatriz Queiroz dos Santos, Victor Dellevedove Cruz, João Vitor Monteiro Berbert, Bruna Carolina Gonçalves, Galdino Andrade, Eliana Carolina Vespero, Gerson Nakazato, Renata Katsuko Takayama Kobayashi

**Affiliations:** ^1^Department of Microbiology, Biological Sciences Center, State University of Londrina, Londrina, Brazil; ^2^Department of Preventive Veterinary Medicine, Avian Medicine Laboratory, State University of Londrina, Londrina, Brazil; ^3^Department of Pathology, Clinical Analysis and Toxicology, Health Sciences Center, State University of Londrina, Londrina, Brazil

**Keywords:** *bla*_*CTX–M–55*_ gene, *fosA3* gene, broiler chicken farms, multidrug resistance, fosfomycin, longitudinal monitoring, ESBL (extended spectrum beta lactamase)

## Abstract

Considering the worrying emergence of multidrug resistance, including in animal husbandry and especially in food-producing animals, the need to detect antimicrobial resistance strains in poultry environments is relevant, mainly considering a One Health approach. Thus, this study aimed to conduct longitudinal monitoring of antimicrobial resistance in broiler chicken farms, with an emphasis on evaluating the frequency of resistance to fosfomycin and β-lactams. *Escherichia coli* was isolated from broiler chicken farms (cloacal swabs, meconium, poultry feed, water, poultry litter, and *Alphitobius diaperinus*) in northern Paraná from 2019 to 2020 during three periods: the first period (1st days of life), the second period (20th to 25th days of life), and third period (40th to 42nd days of life). Antibiogram tests and the detection of phenotypic extended-spectrum β-lactamase (ESBL) were performed, and they were confirmed by seaching for genes from the *bla*_*CTX–M*_ group. The other resistance genes searched were *mcr-1* and *fosA3*. Some ESBL *bla*_*CTX–M–*1_ group strains were selected for ESBL identification by sequencing and enterobacterial repetitive intergenic consensus-polymerase chain reaction analysis. To determine the transferability of the *bla*_*CTX–M–*1–_ and *fosA3*-carrying plasmids, strains were subjected to conjugation experiments. A total of 507 *E. coli* were analyzed: 360 from cloacal swabs, 24 from meconium samples, 3 from poultry feed samples, 18 from water samples, 69 from poultry litter samples, and 33 from *A. diaperinus* samples. Among the strain isolate, 80% (406/507) were multidrug-resistant (MDR), and 51% (260/507) were ESBL-positive, with the *bla*_*CTX–M–*1_ group being the most frequent. For the *fosA3* gene, 68% (344/507) of the strains isolated were positive, deserves to be highlighted *E. coli* isolated from day-old chickens (OR 6.34, CI 2.34–17.17), when compared with strains isolated from other origins (poultry litter, *A. diaperinus*, water, and poultry feed). This work alerts us to the high frequency of the *fosA3* gene correlated with the CTX-M-1 group (OR 3.57, CI 95% 2.7–4.72, *p* < 0.05), especially the *bla*_*CTX–M–*55_ gene, in broiler chickens. This profile was observed mainly in day-old chicken, with a high percentage of *E. coli* that were MDR. The findings emphasize the importance of conducting longitudinal monitoring to detect the primary risk points during poultry production.

## Introduction

Antimicrobial resistance is a global public health threat, as infections with multiresistant bacteria are predicted to become the leading cause of death 30 years from now (O’Neill, 2014; European Commission, 2018). Approximately 33,000 deaths/year are estimated to occur in the European Union due to bacterial resistance to antibiotics (Cassini et al., 2019). Currently, strains with a high resistance profile (Avershina et al., 2021a,b; Zalewska et al., 2021), which could be aggravated by the increased use of antimicrobials during the SARS Cov-2 pandemic, are being increasingly detected (Abelenda-Alonso et al., 2020; Huttner et al., 2020; Lobie et al., 2021; Nieuwlaat et al., 2021).

The One Health concept allows for a greater understanding of the scope of this issue (Thanner et al., 2016; Aslam et al., 2021; Gazal et al., 2021), given the connection that exists between human, animal, and environmental health (Aslam et al., 2018; Aarestrup et al., 2021). Through this, the World Health Organization (WHO), together with the United Nations Food and Agriculture Organization and the World Organization for Animal Health, have promoted the monitoring and surveillance of resistance to antimicrobials, aiming to detect the possible causes and main points responsible for the selection of multiresistant strains (Poirel et al., 2018). Therefore, establishing programs to monitor resistance and control the use of antimicrobials are strategies that can minimize the rapid spread of antimicrobial resistance genes (Schnall et al., 2019).

Some studies have already shown that the poultry environment (Cunha et al., 2017; Saharan et al., 2020; Gazal et al., 2021) and its products (Koga et al., 2015; Davis et al., 2018; Cyoia et al., 2019) are responsible for harboring and transmitting multidrug-resistant (MDR) strains that produce extended-spectrum β-lactamase (ESBL) or other mechanisms with resistance to antimicrobials, that are considered the last resort in human medicine, such as fosfomycin and colistin (WHO, 2018, 2019).

Among the microorganisms present in the poultry environment, *Escherichia coli* stands out and is even considered a bioindicator of antimicrobial resistance (Poirel et al., 2018; Manges et al., 2019; Büdel et al., 2020). MDR strains facilitate the spread of resistance genes and enhance the phenomenon of antimicrobial resistance (Cazares et al., 2020). Among the genes that encode ESBL, the CTX-M group is predominant and the most widespread (Paterson and Bonomo, 2005; Laube et al., 2013; De Oliveira et al., 2020; Song et al., 2020). The *bla*_*CTX–M–*1_ group is the most prevalent gene detected from the CTX-M group, and the CTX-M-55 enzyme has already been described in animal production in countries from Europe, China, and Brazil (Cunha et al., 2017; Lukman et al., 2017; Lupo et al., 2018; Park et al., 2019; Gazal et al., 2021; Lay et al., 2021).

Fosfomycin is used in human medicine in cases of urinary tract infections caused by MDR strains in Brazil (Huttner et al., 2018; Camposda et al., 2020) and cases of bacterial prostatitis (Kwan and Beahm, 2020), with the *fosA3* gene being the most frequently associated with fosfomycin resistance.

Brazil is the world’s largest exporter and the second-largest chicken meat producer. Paraná is the most productive state, and when its production is added to the other states of the southern region of Brazil, it represents 60% of the country’s chicken meat production (Aquino, 2021). In the state, the broiler breeding system works by integrators. Companies provide food, birds, and technical assistance, and farmers provide labor and installations, such as poultry sheds (Frost et al., 2003; Arikan et al., 2017). Thus, common poultry nuclei supply day-old broilers to large geographic regions, constituting the broiler production pyramid (Zurfluh et al., 2014).

Due to high productivity, there is heterogeneity in the size of the farms, the type of poultry sheds, and the number of birds housed, with an average of 14 birds/m^2^ (Frost et al., 2003; Arikan et al., 2017; De Oliveira Sidinei et al., 2021).

Therefore, this study aimed to conduct longitudinal monitoring of antimicrobial resistance in broiler chicken farms, emphasizing the frequency of resistance to fosfomycin and β-lactams.

## Materials and Methods

This project was approved by the Ethics Committee on the Use of Animals of the State University of Londrina – CEUA/UEL, processing number 13142.2019.51.

### Poultry Farm Characteristics

Samples (cloacal swabs, meconium, water, poultry feed, poultry litter, and *Alphitobius diaperinus*) were collected from four broiler farms in the northern region of Paraná – from March 2019 to July 2020, no farms were sampled simultaneously.

The all-in/all-out system was adopted in all farms evaluated, with an interval between batches of 7 to 12 days and a housing capacity from 10,000 to 30,000 chickens. Some sheds were technified (dark house), and others were conventional sheds. The poultry litter (composed of shavings) was reused for up to seven cycles, with fermentation conducted by canvas covering between them (De Oliveira Sidinei et al., 2021).

The animals evaluated came from three different hatcheries, but all were from the same agroindustry.

All farms adopted the use of organic acids in water within 20–25 days of life (dol), and on farms 1, 2, and 3, the supply of antimicrobials was reported, such as tiamulin (before the prohibition of its use) and enrofloxacin (EN; Brasil, 2020).

Regarding water, all the farms provided chlorinated water (1–3 ppm), and the feed was provided by the integrator company, respecting the stages of chicken rearing.

### Poultry Farm Samples

Sample collections (cloacal swabs, meconium, water, poultry feed, poultry litter, and *A. diaperinus*) were collected at three different periods: the first period (1st dol or day-old), the second period (20th to 25th dol), and the third period (40th to 42nd dol).

The cloacal swabs were collected on the first day of life before housing the birds, and meconium was collected from the bottom of the transport boxes. Thirty cloacal swabs were collected per farm, placed in Cary Blair medium (Absorb), and sent under refrigeration for processing. Swabs (*n* = 30) were grouped into five pools, totaling 60 samples.

From the environment, samples were collected from poultry litter (*n* = 12) with sterile boot swabs (Brasil, 2016) using two pairs of props, each sampling 50% of the shed. Feed samples (2 kg/silo per moment, totaling 12 samples) and *A. diaperinus* (100 adult beetles per house, totaling 12 samples) were collected. Water samples were collected at the beginning (initial water) and end (final water) of the drinking fountain line for a total of 24 samples. In total, 124 samples of materials from different sources were processed.

### Processing and Selection of the Isolates

The water samples were analyzed by a multiple-tube fermentation technique according to Eckner (1998) and (Brasil, 2013). After incubation in buffered peptone water, at 37^°^C for 18–24 h, the other samples were seeded on MacConkey agar without supplementation (MC) and supplemented with the antimicrobials, ciprofloxacin (CIP), cefotaxime (CTX), and ciprofloxacin + cefotaxime (CIP/CTX), at a final concentration of 8 μg/ml. The lactose-fermenting colonies were subjected to identification by biochemical screening using triple-sugar iron agar, indole production, Simmons citrate, urease production, lysine decarboxylation, and sorbitol and cellobiose fermentation tests (Merck, Darmstadt, Alemanha; Awogbemi et al., 2018; Moeinizadeh and Shaheli, 2021; Thomrongsuwannakij et al., 2021).

Up to three *E. coli* colonies were selected from each culture medium and stored at -20^°^C in brain heart infusion broth (Himedia Laboratories Pvt. Ltd., Mumbai, Índia) supplemented with 20% glycerol until processing. For the subsequent analyses, six bacterial isolates from each origin were used, per farm, per period.

### Antimicrobial Resistance and Extended-Spectrum β-Lactamase Production

Antimicrobial sensitivity was determined using the disk diffusion method, following the Clinical and Laboratory Standards Institute (CLSI, 2018). Nineteen antimicrobials belonging to seven different classes were used, β-lactams: amoxicillin-clavulanic acid (AMC, 10/20 μg), ampicillin (AMP, 10 μg), cefazolin (CFZ, 30 μg), cefoxitin, (CFO, 30 μg), ceftiofur (CTF, 30 μg), ceftriaxone (CRO, 30 μg), ceftazidime (CAZ, 30 μg), CTX (30 μg), CRO (30 μg), aztreonam (ATM, 30 μg), and imipenem (IPM, 30 μg); quinolones: CIP (5 μg), EN (10 μg), and nalidixic acid (NAL, 30 μg); sulfonamides: sulfamethoxazol + trimethoprim (SXT, 1.25/23.75 μg); tetracyclines: tetracycline (TET, 30 μg); aminoglycosides: gentamicin (CN, 10 μg); amphenicols: chloramphenicol (C, 30 μg); and fosfomycin: fosfomycin/trometamol (FOT, 200 μg; Oxoid Ltd., Basingstoke, Hants, Reino Unido, United Kingdom). *E. coli* ATCC 25922 was used as a standard control for the antibiogram test. The results were interpreted according to CLSI (2019) and BRCAST (2019) except for CTF and EN, which followed CLSI (2008).

For ESBL detection, the double synergism technique was performed (Jarlier et al., 1988) with an AMC disk (10/20 μg), placed at 20 mm disks of ATM (30 μg), CAZ (30 μg), CTF (30 μg), and cefepime (FEP, 30 μg).

### Antimicrobial Resistance Survey

Bacterial DNA was extracted using the Pure Link Genomic DNA Mini Kit (Invitrogen).

We searched for genes that conferred resistance to β-lactams (*bla*_*CTX–M–*1_, *bla*_*CTX–M–*2_, *bla*_*CTX–M–*8_, *bla*_*CTX–M–*9_, and *bla*_*CTX–M–*25_; Woodford et al., 2006), colistin resistance coding genes (*mcr-1*; Liu et al., 2016), and fosfomycin (*fosA3*; Sato et al., 2013).

All PCR amplicons were visualized on 1.5% agarose gels stained with GelRed (Biotium, Hayward, CA, United States). After gel electrophoresis, the images were captured using Image Capture Systems (LPixImageHE).

### Analysis of the Genetic Similarity Profile by Enterobacterial Repetitive Intergenic Consensus-Polymerase Chain Reaction

The genetic similarity profile was evaluated by enterobacterial repetitive intergenic consensus-polymerase chain reaction (ERIC-PCR) analysis, in accordance with Versalovic et al. (1991). The PCR products were subjected to 2% agarose gel electrophoresis for 4 h in TBE buffer, stained with ethidium bromide (0.5 μg/mL), and visualized in a UV transilluminator (Vilbert Loumart). The similarity dendrogram was constructed using Gel J 2.0 software (Heras et al., 2015), using the unweighted pair group method with arithmetic mean (UPGMA) and the data similarity coefficient for cluster analysis (Jaccard), with a tolerance index of 1.0. The standard cutoff level to define the clusters was 85% (Daga et al., 2019).

### Sequencing

Sequencing was performed from the PCR product amplified for IS*Ecp* (Zurita et al., 2016). When negative for this insertion sequence, the amplified product for the gene using *bla*_*CTX–M–*1_ (Fei Tian et al., 2011; Cantón et al., 2012; Dierikx et al., 2013) was characterized for bidirectional Sanger sequencing on ABI-PRISM 3500 XL (Applied Biosystems) following the manufacturer’s recommendations. For the interpretation and alignment of the sequencing results, Chromas was used to evaluate the electropherogram, and ClustalW was used to achieve alignment of the sequences. Using BLAST, the sequences were compared with the NCBI database.

### Conjugation Experiment

Conjugation was performed to assess the horizontal transfer capacity of the *bla*_*CTX–M–*55_ gene and *fosA3* (Gonçalves et al., 2021). Two *E. coli bla*_*CTX–M–*55_ and *fosA3* from Group B1 (negative for *chuA* and *YjaA* and positive for TspE4 *and ArpA*) were used as donors, and one *E. coli* from Group D (negative for TspE4 *and YjaA* and positive for *chuA and ArpA*) resistant to gentamicin was used as the recipient. All isolates were from day-old chickens (Clermont et al., 2013).

### Statistical Evaluation

Statistical analysis was performed using R version 3.5.1. To assess the relationship between the studied variables, multivariate logistic regression analysis was used. The odds ratio (OD) calculation of the prediction model (α = 5%) was used to identify the set of information that best explained the relationship of risk factors associated with the occurrence of *fosA3-* and ESBL-producing *E. coli* (Menard, 2002).

## Results

### *Escherichia coli* Phenotypic and Genotypic Profile

A total of 507 *E. coli* were isolated: 360 from cloacal swabs, 24 from meconium, 3 from poultry feed, 18 from water, 69 from poultry litter, and 33 from *A. diaperinus* (Table 1).

**TABLE 1:** Number of *Escherichia coli* isolates by periods and samples.

Periods	Farms	Poultry farm 1	Poultry farm 2	Poultry farm 3	Poultry farm 4	Total
		
	Samples	*(Number of isolates)*				
First period (1st day of life)	*Meconium*	6	6	6	6	24
	*First pool of cloacal swab*	6	6	6	6	24
	*Second pool of cloacal swab*	6	6	6	6	24
	*The third pool of cloacal swab*	6	6	6	6	24
	*The fourth pool of cloacal swab*	6	6	6	6	24
	*The fifth pool of cloacal swab*	6	6	6	6	24
	*Poultry feed*	0	0	3	0	3
	*Initial water*	0	3	0	3	6
	*Final water*	0	3	3	3	9
	*Poultry litter*	3	6	6	6	21
	*Alphitobius diaperinus*	NA	NA	NA	NA	0
Second period (20th to 25th days of life)	*First pool of cloacal swab*	6	6	6	6	24
	*Second pool of cloacal swab*	6	6	6	6	24
	*The third pool of cloacal swab*	6	6	6	6	24
	*The fourth pool of cloacal swab*	6	6	6	6	24
	*The fifth pool of cloacal swab*	6	6	6	6	24
	*Poultry feed*	0	0	0	0	0
	*Initial water*	0	0	0	0	0
	*Final water*	0	0	0	0	0
	*Poultry litter*	6	6	6	6	24
	*Alphitobius diaperinus*	0	6	0	3	9
Third period (40th to 42nd days of life)	*First pool of cloacal swab*	6	6	6	6	24
	*Second pool of cloacal swab*	6	6	6	6	24
	*The third pool of cloacal swab*	6	6	6	6	24
	*The fourth pool of cloacal swab*	6	6	6	6	24
	*The fifth pool of cloacal swab*	6	6	6	6	24
	*Poultry feed*	0	0	0	0	0
	*Initial water*	0	0	0	0	0
	*Final water*	0	0	3	0	3
	*Poultry litter*	6	6	6	6	24
	*Alphitobius diaperinus*	6	6	6	6	24

Among the 507 *E. coli* isolated, fosfomycin (41% – 202/507) and AMP (68% – 347/507) are the antimicrobials that deserve the most attention due to their high percentage of resistance. Therefore, more than 80.1% (406/507) were MDR, and more than 51% (260/507) were ESBL producers. Regarding resistance to β-lactams, the *bla*_*CTX–M–*1_ group, was present in 40% (202/507) of the strains, the most frequent *bla*_*CTX–M*_ group, followed by the *bla*_*CTX–M–*2_ group at 17% (86/507; Table 2). The strains that presented some gene from the *bla*_*CTX–M*_ group had greater resistance to CAZ (98.9%, 268/271), CFZ (94.5%, 256/271), and AMP (92.3%, 250/271).

**TABLE 2:** Percentage (%) of isolates with MDR, ESBL, *bla*_*CTX–M–*1_ group, *bla*_*CTX–M–*2_ group, or *fosA3* gene from the cloacal swab, meconium, poultry litter, and *A. diaperinus* samples, per period.

	Periods/Samples	First period (1st days of life) %	Second period (20th to 25th days of life) %	Third period (40th to 42nd days of life) %
		
		(Positive samples/total samples)		
MDR	Cloacal Swab	83.3 (100/120)	90 (108/120)	81.7 (98/120)
	Meconium	83.3 (20/24)	Not sampled	Not sampled
	Poultry litter	81 (17/21)	83.3 (20/24)	79.2 (19/24)
	*A. diaperinus*	Not sampled	55.6 (5/9)	75 (18/24)
ESBL	Cloacal Swab	59.2 (71/120)	54.2 (65/120)	51.7 (62/120)
	Meconium	54.2 (13/24)	Not sampled	Not sampled
	Poultry litter	42.9 (2/21)	58.3 (14/24)	50 (12/24)
	*A. diaperinus*	Not sampled	33.3 (3/9)	45.8 (11/24)
*bla*_*CTX–M–*1_	Cloacal Swab	55 (66/120)	48.3 (58/120)	24.2 (29/120)
	Meconium	37.5 (9/24)	Not sampled	Not sampled
	Poultry litter	38.1 (8/21)	45.8 (11/24)	50 (12/24)
	*A. diaperinus*	Not sampled	66.7 (6/9)	12.5 (3/24)
*bla*_*CTX–M–*2_	Cloacal Swab	21.7 (21/120)	15.8 (19/120)	17.5 (21/120)
	Meconium	8.3 (2/24)	Not sampled	Not sampled
	Poultry litter	23.8 (5/21)	12.5 (3/24)	8.3 (2/24)
	*A. diaperinus*	Not sampled	11.1 (1/9)	29.2 (7/24)
*fosA3*	Cloacal Swab	78 (92/120)	74.2 (89/120)	61.7 (74/120)
	Meconium	87.5 (21/24)	Not sampled	Not sampled
	Poultry litter	66.7 (14/21)	75 (18/24)	66.7 (16/24)
	*A. diaperinus*	Not sampled	66.7 (6/9)	58.3 (14/24)

Comparing the resistance profiles of the strains, no significant difference was observed between the sampled periods regarding the presence of MDR and *fosA3* and the occurrence of ESBL. For the simultaneous presence of the *fosA3* and *bla*_*CTX–M*_ genes, the strain that had *fosA3* was 3.57 (CI 2.7–4.72) times more likely to also have a gene in the *bla*_*CTX–M–*1_ group.

### *Escherichia coli* Isolated From Cloacal Swab and Meconium Samples

Twenty-four *E. coli* samples from meconium and 360 *E. coli* samples from cloacal swabs were isolated, totaling 384 *E. coli* from chickens (Table 1). On all farms surveyed and, in all periods, *E. coli* ESBL-production was detected.

**FIGURE 1:**
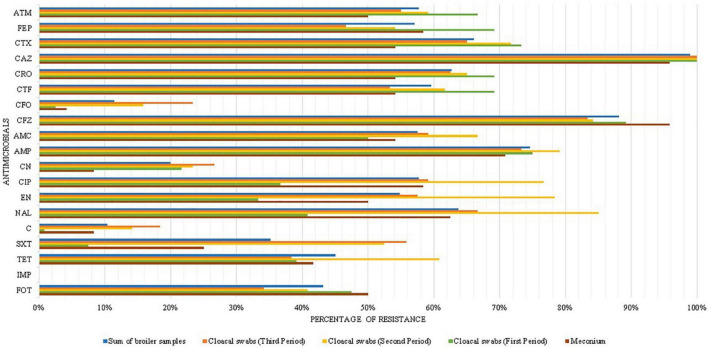
Phenotypic resistance profile of broiler samples by periods. FOT, Fosfomycin-trometamol; TET, tetracycline; SXT, trimethoprim-sulfamethoxazole; C, chloramphenicol; CN, gentamicin; CIP, ciprofloxacin; NAL, nalidixic acid; EN, enrofloxacin; AMC, amoxicillin-clavulanic acid; AMP, ampicillin; CFZ, cefazolin; CFO, cefoxitin; CTF, ceftiofur; CRO, ceftriaxone; CAZ, ceftazidime; CTX, cefotaxime; FEP, cefepime; ATM, aztreonam; and IMP, imipenem.

In the first period, *E. coli* resistant to CTX (70% – 101/144) and fosfomycin (47.9% – 69/144) was isolated from meconium and cloacal swab samples, and the first period was 1.64 (CI 1.01–2.65) times more likely to harbor the *fosA3* gene, than other periods. In the second period, the highest percentage of MDR was observed (90% – 108/120). In the third period, there was a decrease in strains resistant to some antimicrobials, such as fosfomycin (34% – 41/120), quinolones, and third and fourth generation cephalosporins, and it was the period with the lowest number of strains harboring the *fosA3* gene (OR 0.49, CI 0.31–0.77), compared to the first and second periods (Figure 1).

Strains isolated from chickens (meconium + cloacal swabs) were 1.62 (CI 1.19–2.2) times more likely to be ESBL producers and present the *fosA3* gene than the other samples. Cloacal swab samples were 2.53 (CI 1.61–3.97) times more likely to be resistant to three or more classes of antimicrobials.

None of the strains isolated were positive for the *mcr-1* gene or *bla*_*CTX–M–*9_ and *bla*_*CTX–M–*25_ groups. However, five strains isolated from cloacal swabs had the *bla*_*CTX–M–*8_ group, and they were from different periods and cloacal swab samples but belonged to the same farm.

### *Escherichia coli* Isolated From Poultry Litter and *A. diaperinus* Samples

**FIGURE 2:**
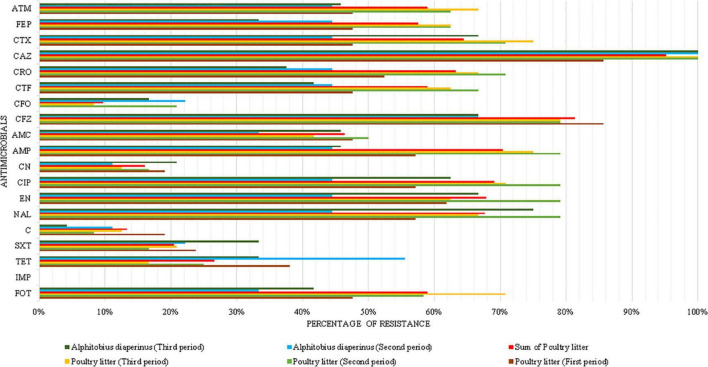
Phenotypic resistance profile of poultry litter and *A. diaperinus* by periods. FOT, Fosfomycin-trometamol; TET, tetracycline; SXT, trimethoprim-sulfamethoxazole; C, chloramphenicol; CN, gentamicin; CIP, ciprofloxacin; NAL, nalidixic acid; EN, enrofloxacin; AMC, amoxicillin-clavulanic acid; AMP, ampicillin; CFZ, cefazolin; CFO, cefoxitin; CTF, ceftiofur; CRO, ceftriaxone; CAZ, ceftazidime; CTX, cefotaxime; FEP, cefepime; ATM, aztreonam; and IMP, imipenem.

A total of 69 *E. coli* isolated from poultry litter were obtained (Table 1). In one of the farms, no strains isolated from poultry litter were resistant to CIP or CTX in the first period. In the second period, the frequency of the isolation of ESBL-producing *E. coli* was higher than that of the others, so the MDR profile prevailed in this evaluation period (83.3% – 20/24). Notably, a gradual increase in resistance to fosfomycin and CTX was found from the first period to the second (Figure 2). Strains isolated from poultry litter were more likely to be ESBL producers (OR 2.07, CI 1.31–3.27); additionally, they were more likely (OR 1.64, CI 1.08–2.48) to have the *fosA3* and *bla*_*CTX–M–*1_ genes than other samples.

Regarding, only one strain was positive (5% – 1/21) to the *mcr-1* gene, and it came from the first period; in this strain, the presence of the *fosA3* and *bla*_*CTX–M–*2_ genes was also observed (Table 2).

A total of 33 *E. coli* strains isolated from *A. diaperinus* were collected in the second and third periods (Table 1). The highest percentage of MDR- and ESBL-producing isolates was observed in the third period (Table 2). *A. diaperinus* was not collected in the first period since these beetles were not present on the farms.

### *Escherichia coli* Isolated From Poultry Feed and Water Samples

In the feed samples, *E. coli* were isolated on only one farm (farm 3 – Table 1), which showed 100% resistance to NAL, CIP, and CFZ, and were sensitive to some antimicrobials, such as fosfomycin. Poultry feed-isolated strains tended not to be MDR-, ESBL- or *fosA3*-positive (OR < 0.01).

Regarding the water samples, 18 strains were obtained, and a more significant recovery of *E. coli* occurred in the first period (Table 1).

None of the strains isolated from the water were MDR or ESBL positive, nor did they present any resistance genes studied. Strains isolated from start water and end water tended not to be MDR-, ESBL-, or *fosA3*-positive (OR < 0.01).

### Enterobacterial Repetitive Intergenic Consensus Sequence (ERIC-PCR) and Sequencing

Forty-three bacterial isolates from poultry litter, cloacal swabs, and meconium, positive for the *bla*_*CTX–M–*1_ and *fosA3* genes, were selected for ERIC-PCR analysis and *bla*_*CTX–M–*1_ sequencing. The sequenced isolates carried the enzyme *bla*_*CTX–M–*55_.

**FIGURE 3:**
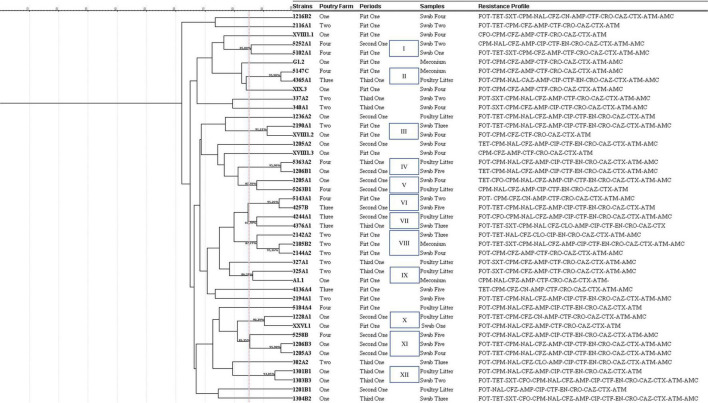
Dendrogram with the characteristics of *E. coli* harboring *fosA3*/CTX-M-55 by ERIC-PCR. FOT, Fosfomycin-trometamol; TET, tetracycline; SXT, trimethoprim-sulfamethoxazole; C, chloramphenicol; CN, gentamicin; CIP, ciprofloxacin; NAL, nalidixic acid; EN, enrofloxacin; AMC, amoxicillin-clavulanic acid; AMP, ampicillin; CFZ, cefazolin; CFO, cefoxitin; CTF, ceftiofur; CRO, ceftriaxone; CAZ, ceftazidime; CTX, cefotaxime; FEP, cefepime; ATM, aztreonam; and IMP, imipenem.

Regarding ERIC-PCR, ten clonal groups with more than 85% similarity were observed. Some of these groups were represented by *E. coli* isolated from cloacal swabs in the first periods but from different farms (clonal group III), *E. coli* isolated from the same farm but at different periods (clonal group I), and *E. coli* isolated from different farms, periods, and sources (clonal groups I, VI, VIII, and IX), as shown in Figure 3.

### Conjugation Experiment Results

Two transconjugants were obtained in the conjugation assay; these isolates belonged to phylogenetic group D, confirming that they are the recipient strains and were positive for the *bla*_*CTX–M–*55_ and *fosA3* groups, demonstrating the horizontal transfer capacity of these genes.

## Discussion

Emerging resistance to antimicrobials is a concern and needs to be investigated. According to PanBr-Agro, broiler farms are important places for monitoring resistance to antimicrobials (Brasil, 2018) due to the sanitary challenges in the poultry production system and the consequent use of antimicrobials for different purposes. Thus, poultry production is one of the segments responsible for the selection of isolates resistant to antimicrobials (Cuong et al., 2019; Hedman et al., 2020; Zalewska et al., 2021), and the control of some antimicrobials can minimize zoonotic risks of multiresistant transmission bacteria from animals to humans (Magnusson, 2020). Our study carried out a longitudinal monitoring, researching multiresistant bacteria from different samples in three life periods of broilers, to investigate possible risk points in the spread of antimicrobial resistance.

Our results demonstrate that the main critical point for the spread of antimicrobial resistance in broiler chicken farms was the chickens, which arrived colonized with *E. coli* harboring the *fosA3* and *bla*_*CTX–M–*1_ genes, especially the *bla*_*CTX–M–*55_. We detected 68% (344/507) of the isolates positive for the *fosA3* gene. Our *fosA3* positive strains were from cloacal swab, meconium, *A. diaperinus*, and poultry litter samples.

According to the WHO, fosfomycin is in the AWaRe classification group, and it acts against strains resistant to carbapenems (Martirosov and Lodise, 2016; Pauwels et al., 2021). Cottell and Webber (2017) analyzed 676 *E. coli* isolated from urine in a hospital in the United Kingdom, and 12% were resistant to fosfomycin. The authors demonstrated that *E. coli* isolated from infections in humans have a low resistance to fosfomycin and highlighted the importance of keeping this antimicrobial for human use only. Derington et al. (2020) tested fosfomycin as a therapeutic option for treating complicated urinary tract infections, leading to the resolution of the evaluated conditions. Based on these results, they, and other authors report that fosfomycin is an essential human therapeutic option (Wachino et al., 2010; Neuner et al., 2012; Jean et al., 2016; Cao et al., 2017; Abbott et al., 2020; Loras et al., 2020; Seok et al., 2020), against urinary infections for example, the second most important bacterial infection affecting humans (Moeinizadeh and Shaheli, 2021).

It is an antimicrobial that should only be used against multiresistant microorganisms (WHO, 2019); therefore, its use should be restricted to human medicine. Notably, Brazil does not have a ban on using this antimicrobial in animal production, being a country that uses fosfomycin in poultry production (Roth et al., 2019). The use of antimicrobials leads to selective pressure and consequently the selection of resistant strains (WHO, 2018, 2019); thus, the monitoring and surveillance of antimicrobials in animal production should promote the rational use of the same.

We report that both meconium and cloacal swab samples from day-old chickens (first period) had a 1.64 (CI 1.01–2.65) times greater chance of harboring the *fosA3* gene than those from other periods. Vertical transmission can occur at any point in poultry production before the chickens reach the farms. In a longitudinal study, Poulsen et al. (2017) demonstrated that broiler breeders could transmit microorganisms to broilers. Although vertical transmission of *E. coli* to day-old chickens has already been shown by other authors (Lu et al., 2003; Dierikx et al., 2013; Fischer et al., 2014; Zurfluh et al., 2014; Osman et al., 2018; Apostolakos et al., 2019, 2020; Dame-Korevaar et al., 2019, Dame-Korevaar et al., 2020; Zhao et al., 2019), this is the first report of the presence of fosfomycin-resistant *E. coli* in day-old chickens.

Enterobacterial repetitive intergenic consensus-polymerase chain reaction detected clonal groups formed by strains from different farms isolated from day-old chickens (first period) and groups with *E. coli* isolated from the same farm but at different periods (clonal group I), suggesting possible vertical transmission and the perpetuation of *fosA3*/CTX-M-55 samples, respectively. This finding serves as a warning about the potential spread of resistance within the broiler production chain.

We also showed that strains isolated from poultry litter were more likely to be ESBL producers (OR 2.07, CI 1.31–3.27) than other samples, an important point in disseminating and perpetuating ESBL strains in poultry farming. Curiously in our research, poultry litter did not have the high number of resistance when compared with day-old chicken sample. The use of the same poultry litter in subsequent production cycles, when subjected to a sanitary vacuum and a good fermentation process, may result in a low microbial load (Wei et al., 2013; Vieira et al., 2015; Waziri and Kaltungo, 2017; Gurmessa et al., 2021; Moffo et al., 2021; Yévenes et al., 2021). In Brazil, poultry litter is reused for more than one consecutive batch, and this practice is allowed if sanitary problems have not occurred with the batches housed on the poultry litter to be reused (Waziri and Kaltungo, 2017).

Gazal et al. (2021) sampled broiler farms and found that the main sources of ESBL dissemination were poultry litter and *Alphitobius* sp. samples, which were detected from day one in farms sampled. Our study did not isolate MDR and ESBL-producing *E. coli* from all farms in the first period from poultry litter. One of the broiler farms analyzed reported having performed efficient poultry litter management, demonstrated by the low resistance profile of the strains isolated. In this and in other poultry farms it was also possible to observe a reduction or elimination of *A. diaperinus* in the first periods, possibly due to the poultry litter treatment process carried out between batches. Siller et al. (2020) reported that even when poultry litter undergoes a fermentation process, other events can interfere with the number of microorganisms present, regardless of how many times the litter has already been used. Therefore, it is essential to point out that poultry litter is indeed a sample that presents a potential risk factor inside poultry houses.

In our study, when we subjected *E. coli* strains harboring *fosA3* and *_*bla*_*_*CTX–M–*55_ to conjugation experiments with a strain sensitive to fosfomycin and CTX, we observed that they were able to transmit both resistance genes. With the spread of these resistant strains, an increase in therapeutic failures in treating ESBL-producing microorganisms is expected. The use of fosfomycin risks the selection of ESBL coproducers since CTX-M, and *fosA3* genes have already been confirmed to colocalize on plasmids (Sarker et al., 2019). Another important point to highlight about conjugation is that the recipient strain belongs to phylogenetic group D, and the donor strain belongs to phylogenetic Group B1, which demonstrates an exchange of genetic material between commensal microorganisms and microorganisms with potential pathogenicity (Clermont et al., 2013).

Regarding the *E. coli* isolated from water and poultry feed, these strains were not identified as MDR microorganisms or ESBL producers. This finding characterizes the samples as not critical points for the spread of antimicrobial resistance in broiler production (Miles et al., 2006; Rossato et al., 2019; Gazal et al., 2021).

In the present work, 4.33% (25/507) of the strains isolated had more than one gene from the *bla*_*CTX–M*_ group, which should be highlighted regardless of the low percentage due to the potential for the development of other enzyme recombinants by these strains in the future (Yin et al., 2020; Leão et al., 2021).

Colistin is an antimicrobial indicated as a last resort to treat infections in humans. The first report of the *mcr-1* gene, one of the genes that confers resistance to this antimicrobial, was in China by Liu et al. (2016), and *mcr-1* has been correlated with the emergence of pandrug-resistant microorganisms (McGann et al., 2016). The *mcr-1* gene is more frequently detected in farm animals than in humans (Elbediwi et al., 2019) but can cause serious infectious diseases such as pyelonephritis in humans (Birgy et al., 2018). Ahmed et al. (2020) found in Bangladesh that 25% (300/1200) of *E. coli* strains isolated from broiler chicken farms were positive for the *mcr-1* gene. Out of 507 *E. coli* isolates, we detected only one positive *mcr-1* gene in our work. This is possibly due to the local prohibition of using this antimicrobial as a growth promoter. This strain is a fourth-generation cephalosporin-resistant, producer of ESBL (*bla*_*CTX–M–*2_ group) and harbors the *fosA3* gene.

We used culture media supplemented with antimicrobials to select isolates in the present work. This technique can favor the selection of strains with a higher resistance profile and more significant gene variability (Ceccarelli et al., 2019). We used preselection, CTX, an indicator of the CTX-M group (Paterson and Bonomo, 2005), and CIP antimicrobial with coselection to the ESBL enzyme (Jiang et al., 2008; Hassan et al., 2012; Seo and Lee, 2019). In this way, we were able to detect strains with a high resistance profile, avoiding underreporting of the antimicrobial-resistant profile.

Hence, in this work, a high frequency of *E. coli* harboring the *fosA3*/CTX-M-55 strains was detected in poultry farming, from day-old chickens to preslaughter chickens, and in different samples (cloacal swab + meconium, chicken litter, and beetles), with these genes transferable by conjugation. Therefore, considering the importance of fosfomycin to human medicine and the fact that Brazil is one of the largest exporters of chicken meat in the world (ABPA, 2021) and one of the few countries that use this antimicrobial in poultry production, we reinforce the need to ban its use in the poultry sector.

## Conclusion

In conclusion, this study demonstrated that the spread of *fosA3*-mediated fosfomycin resistance was correlated with the presence of the CTX-M-1 group, especially the *bla*_*CTX–M–*55_ gene, in broiler chickens. This profile was observed mainly in day-old chickens, with a high profile of *E. coli* strains multidrug resistant to antimicrobials. The findings emphasize the importance of conducting longitudinal monitoring to detect the main risk points in poultry production and thus intervene, prevent drug resistance, and promote the rational use of antimicrobials.

## Data Availability Statement

The datasets presented in this study can be found in online repositories. The names of the repository/repositories and accession number(s) can be found below: NCBI GenBank – OM326827–OM326869.

## Ethics Statement

The animal study was reviewed and approved by Animal Ethics Committee of State University of Londrina (CEUA/UEL). Processing number: 13142.2019.51.

## Author Contributions

MM-C contributed to developing experimental research, data analysis, and writing the manuscript. AB, LG, LJ, MdS, MS, BQ, VC, JB, GA, and BG contributed to the development of experimental research. EN contributed to the statistical analysis. RK, GN, AB, EV, LG, and GA contributed to and assisted in the design of the work and preparation of the article and critically reviewed the manuscript. All authors have participated in this study and commented on the manuscript.

## Conflict of Interest

The authors declare that the research was conducted in the absence of any commercial or financial relationships that could be construed as a potential conflict of interest.

## Publisher’s Note

All claims expressed in this article are solely those of the authors and do not necessarily represent those of their affiliated organizations, or those of the publisher, the editors and the reviewers. Any product that may be evaluated in this article, or claim that may be made by its manufacturer, is not guaranteed or endorsed by the publisher.
