# Supplementary material for: High-Frequency Detection of fosA3 and blaCTX–M–55 Genes in Escherichia coli From Longitudinal Monitoring in Broiler Chicken Farms
Source: Front Microbiol. 2022 May 18;13:846116. doi: 10.3389/fmicb.2022.846116 (PMC9158547; doi:10.3389/fmicb.2022.846116)
Supplement: Supplementary file 1 [file Table_1.docx]

| **Meconium**  **(n=24)** | **FOT** | **IMP** | **TET** | **SXT** | **C** | **NAL** | **EN** | **CIP** | **CN** | **AMP** | **AMC** | **CFZ** | **CFO** | **CTF** | **CRO** | **CAZ** | **CTX** | **FEP** | **ATM** |
| --- | --- | --- | --- | --- | --- | --- | --- | --- | --- | --- | --- | --- | --- | --- | --- | --- | --- | --- | --- |
| *Number of positives isolates* | 12 | 10 | 0 | 6 | 1 | 14 | 15 | 23 | 2 | 2 | 17 | 14 | 13 | 12 | 13 | 23 | 13 | 12 | 13 |
| *Percentage of resistance* | 50% | 42% | 0% | 25% | 4% | 58% | 63% | 96% | 8% | 8% | 71% | 58% | 54% | 50% | 54% | 96% | 54% | 50% | 54% |
| **Cloacal swabs**  **(First Period)**  **(n=120)** |  |  |  |  |  |  |  |  |  |  |  |  |  |  |  |  |  |  |  |
| *Number of positives isolates* | 57 | 47 | 0 | 9 | 3 | 83 | 49 | 107 | 1 | 26 | 90 | 44 | 83 | 40 | 83 | 120 | 88 | 80 | 60 |
| *Percentage of resistance* | 48% | 39% | 0% | 8% | 3% | 69% | 41% | 89% | 1% | 22% | 75% | 37% | 69% | 33% | 69% | 100% | 73% | 67% | 50% |
| **Cloacal swabs**  **(Second Period)**  **(n=120)** |  |  |  |  |  |  |  |  |  |  |  |  |  |  |  |  |  |  |  |
| *Number of positives isolates* | 49 | 73 | 0 | 63 | 19 | 65 | 102 | 101 | 17 | 28 | 95 | 92 | 74 | 94 | 78 | 120 | 86 | 71 | 80 |
| *Percentage of resistance* | 41% | 61% | 0% | 53% | 16% | 54% | 85% | 84% | 14% | 23% | 79% | 77% | 62% | 78% | 65% | 100% | 72% | 59% | 67% |
| **Cloacal swabs**  **(Third Period)**  **(n=120)** |  |  |  |  |  |  |  |  |  |  |  |  |  |  |  |  |  |  |  |
| *Number of positives isolates* | 41 | 46 | 0 | 67 | 28 | 56 | 80 | 100 | 22 | 32 | 88 | 71 | 64 | 69 | 75 | 120 | 78 | 66 | 71 |
| *Percentage of resistance* | 34% | 38% | 0% | 56% | 23% | 47% | 67% | 83% | 18% | 27% | 73% | 59% | 53% | 58% | 63% | 100% | 65% | 55% | 59% |

**Table 1**: Number of strains isolated from broiler samples (cloacal swabs and meconium), per period, the number of isolates resistance to antimicrobials and the percentage of resistance.

*Fosfomycin-trometamol (FOT), tetracycline (TET), trimethoprim-sulfamethoxazole (SXT), chloramphenicol (C), gentamicin (CN), ciprofloxacin (CIP), nalidixic acid (NAL), enrofloxacin (EN), amoxicillin-clavulanic acid (AMC), ampicillin (AMP), cefazolin (CFZ), cefoxitin (CFO), ceftiofur (CTF), ceftriaxone (CRO), ceftazidime (CAZ), cefotaxime (CTX), cefepime (FEP), aztreonam (ATM), imipenem (IMP).
