# Supplementary material for: High-Frequency Detection of fosA3 and blaCTX–M–55 Genes in Escherichia coli From Longitudinal Monitoring in Broiler Chicken Farms
Source: Front Microbiol. 2022 May 18;13:846116. doi: 10.3389/fmicb.2022.846116 (PMC9158547; doi:10.3389/fmicb.2022.846116)
Supplement: Supplementary file 2 [file Table_2.docx]

| **Poultry litter**  **(First period)**  **(n=21)** | **FOT** | **IMP** | **TET** | **SXT** | **C** | **NAL** | **EN** | **CIP** | **CN** | **AMP** | **AMC** | **CFZ** | **CFO** | **CTF** | **CRO** | **CAZ** | **CTX** | **FEP** | **ATM** |
| --- | --- | --- | --- | --- | --- | --- | --- | --- | --- | --- | --- | --- | --- | --- | --- | --- | --- | --- | --- |
| *Number of positive isolates* | 10 | 8 | 0 | 5 | 0 | 10 | 12 | 18 | 4 | 4 | 12 | 12 | 10 | 13 | 11 | 18 | 10 | 10 | 10 |
| *Percentage of resistance* | 48% | 38% | 0% | 24% | 0% | 48% | 57% | 86% | 19% | 19% | 57% | 57% | 48% | 62% | 52% | 86% | 48% | 48% | 48% |
| **Poultry litter**  **(Second period)**  **(n=24)** |  |  |  |  |  |  |  |  |  |  |  |  |  |  |  |  |  |  |  |
| *Number of positive isolates* | 14 | 6 | 0 | 4 | 5 | 15 | 19 | 19 | 2 | 4 | 19 | 19 | 16 | 19 | 17 | 24 | 17 | 15 | 12 |
| *Percentage of resistance* | 58% | 25% | 0% | 17% | 21% | 63% | 79% | 79% | 8% | 17% | 79% | 79% | 67% | 79% | 71% | 100% | 71% | 63% | 50% |
| **Poultry litter**  **(Third period)**  **(n=24)** |  |  |  |  |  |  |  |  |  |  |  |  |  |  |  |  |  |  |  |
| *Number of positive isolates* | 17 | 4 | 0 | 5 | 2 | 15 | 16 | 19 | 3 | 3 | 18 | 17 | 15 | 15 | 16 | 24 | 18 | 16 | 10 |
| *Percentage of resistance* | 71% | 17% | 0% | 21% | 8% | 63% | 67% | 79% | 13% | 13% | 75% | 71% | 63% | 63% | 67% | 100% | 75% | 67% | 42% |

**Table 2**: Number of strains isolated from Poultry litter, per period, the number of isolates resistance to antimicrobials and the percentage of resistance.

*Fosfomycin-trometamol (FOT), tetracycline (TET), trimethoprim-sulfamethoxazole (SXT), chloramphenicol (C), gentamicin (CN), ciprofloxacin (CIP), nalidixic acid (NAL), enrofloxacin (EN), amoxicillin-clavulanic acid (AMC), ampicillin (AMP), cefazolin (CFZ), cefoxitin (CFO), ceftiofur (CTF), ceftriaxone (CRO), ceftazidime (CAZ), cefotaxime (CTX), cefepime (FEP), aztreonam (ATM), imipenem (IMP).
