# Supplementary material for: High-Frequency Detection of fosA3 and blaCTX–M–55 Genes in Escherichia coli From Longitudinal Monitoring in Broiler Chicken Farms
Source: Front Microbiol. 2022 May 18;13:846116. doi: 10.3389/fmicb.2022.846116 (PMC9158547; doi:10.3389/fmicb.2022.846116)
Supplement: Supplementary file 3 [file Table_3.docx]

| **Alphitobius diaperinus**  **(Second period)**  **(n=9)** | **FOT** | **IMP** | **TET** | **SXT** | **C** | **NAL** | **EN** | **CIP** | **CN** | **AMP** | **AMC** | **CFZ** | **CFO** | **CTF** | **CRO** | **CAZ** | **CTX** | **FEP** | **ATM** |
| --- | --- | --- | --- | --- | --- | --- | --- | --- | --- | --- | --- | --- | --- | --- | --- | --- | --- | --- | --- |
| *Number of isolates positives* | 3 | 5 | 0 | 2 | 2 | 4 | 4 | 6 | 1 | 1 | 4 | 4 | 4 | 4 | 4 | 9 | 4 | 4 | 3 |
| *Percentage of resistance* | 33% | 56% | 0% | 22% | 22% | 44% | 44% | 67% | 11% | 11% | 44% | 44% | 44% | 44% | 44% | 100% | 44% | 44% | 33% |
| **Alphitobius diaperinus**  **(Third period)**  **(n=24)** |  |  |  |  |  |  |  |  |  |  |  |  |  |  |  |  |  |  |  |
| *Number of isolates positives* | 10 | 8 | 0 | 8 | 4 | 8 | 18 | 16 | 1 | 5 | 11 | 15 | 10 | 16 | 9 | 24 | 16 | 11 | 11 |
| *Percentage of resistance* | 48% | 39% | 0% | 8% | 3% | 69% | 41% | 89% | 1% | 22% | 75% | 37% | 69% | 33% | 69% | 100% | 73% | 67% | 50% |

**Table 3**: Number of strains isolated from *Alphitobius diaperinus*, per period, the number of isolates resistance to antimicrobials and the percentage of resistance.

*Fosfomycin-trometamol (FOT), tetracycline (TET), trimethoprim-sulfamethoxazole (SXT), chloramphenicol (C), gentamicin (CN), ciprofloxacin (CIP), nalidixic acid (NAL), enrofloxacin (EN), amoxicillin-clavulanic acid (AMC), ampicillin (AMP), cefazolin (CFZ), cefoxitin (CFO), ceftiofur (CTF), ceftriaxone (CRO), ceftazidime (CAZ), cefotaxime (CTX), cefepime (FEP), aztreonam (ATM), imipenem (IMP).
